# Infestation by *Myzus persicae* Increases Susceptibility of *Brassica napus* cv. “Canard” to *Rhizoctonia solani* AG 2-1

**DOI:** 10.3389/fpls.2018.01903

**Published:** 2018-12-21

**Authors:** Fryni Drizou, Toby J. A. Bruce, Rumiana V. Ray, Neil S. Graham

**Affiliations:** ^1^Division of Plant and Crop Sciences, School of Biosciences, The University of Nottingham, Sutton Bonington Campus, Loughborough, United Kingdom; ^2^School of Life Sciences, Keele University, Keele, United Kingdom

**Keywords:** biotic interactions, oilseed rape, plant signaling, pathogen, aphids

## Abstract

Activation of plant defense pathways can be influenced by the presence of different species of attacking organisms. Understanding the complicated interactions triggering plant defense mechanisms is of great interest as it may allow the development of more effective and sustainable disease control methods. *Myzus persicae* and *Rhizoctonia solani* anastomosis group (AG) 2-1 are two important organisms attacking oilseed rape (OSR), causing disease and reduced yields. At present, is unclear how these two interact with each other and with OSR defenses and therefore the aim of the present study was to gain a better insight into the indirect interaction between aphids and pathogen. In separate experiments, we assessed the effect of AG 2-1 infection on aphid performance, measured as growth rate and population increase and then the effect of aphid infestation on AG 2-1 by quantifying disease and the amount of fungal DNA in plant stems and compost for two OSR varieties, “Canard” and “Temple.” Additionally, we examined the expression of genes related to jasmonic acid (JA) and salicylic acid (SA) defense pathways. There was no significant effect of AG 2-1 infection on *M. persicae* performance. However, aphid infestation in one of the varieties, “Canard,” resulted in significantly increased disease symptoms caused by AG 2-1, although, the amount of fungal DNA was not significantly different between treatments. This meant that “Canard” plants had become more susceptible to the disease. Expression of *LOX3* and *MYC2* was elevated under AG 2-1 treatment but downregulated in plants with both aphids and pathogen. Therefore it seems plausible that alterations in the JA signaling due to aphid infestation resulted in the increased susceptibility to AG 2-1.

## Introduction

Plants are exposed to a variety of attacking organisms aboveground and belowground, including pathogens and herbivorous insects. Plants have coevolved to defend themselves either with constitutive or with more energy-effective inducible defenses, while in response to these, attackers have also evolved counter-defenses (Glazebrook, 2005; Pieterse and Dicke, 2007; Bruce, 2015). The interactions between an attacker and a host plant embrace the recognition of herbivore associated molecular patterns (HAMPs) or pathogen associated molecular patterns (PAMPs) (for herbivorous insects and pathogens, respectively) by the plant which lead to plant triggered immunity (PTI). However, herbivores and pathogens are able to overcome this first layer of plant defenses, by the secretion of effectors and plants respond with a second layer of defenses named effectors triggered immunity (ETI) (Jaouannet et al., 2014; Wang et al., 2014; Bruce, 2015). Chemical defenses and secondary metabolites also have a crucial role in plant defenses (Bruce, 2015). The plant hormones salicylic acid (SA), jasmonic acid (JA), ethylene (ET), and abscisic acid (ABA) are known to play a key role in regulating plant defenses. JA and SA are thought to be the most important, with JA typically activated upon herbivory by chewing insects, wounding and necrotrophic pathogens and SA against biotrophic pathogens and phloem feeding insects (Glazebrook, 2005; Vos et al., 2013). Although SA and JA often act antagonistically, recent studies provide evidence that SA and JA can also act in a synergistic way (Liu et al., 2016) and their activation is highly dependent on the nature of the attacker (feeding guild of herbivore and lifestyle of the pathogen) as well as the plant species (Glazebrook, 2005; Bari and Jones, 2009).

More complex interactions can take place when different attackers share the same host because attackers interact with each other indirectly by inducing changes in their shared host (Bruce and Pickett, 2007; Schultz et al., 2013; Lazebnik et al., 2014; Zhu et al., 2014; Drakulic et al., 2017). For example, aphid development was negatively affected when *Aphis fabae* fed on *Vicia faba* plants infected with *Botrytis cinerea* (a necrotroph) whereas when plants were infected with *Uromyces viciae-fabae* (a biotroph) aphids had better performance or performed equally well as on the control plants (Al-Naemi and Hatcher, 2013). These results were related to induced alterations in nitrogen content after pathogen infection and the authors also speculated that Botrytis-induced JA defenses and *U. viciae fabae*- induced SA defenses played a role (Al-Naemi and Hatcher, 2013). When *Sitobion avenae* aphids and *Fusarium graminearum* share wheat as a host plant, disease severity was increased but aphid survival was decreased (Drakulic et al., 2015). Pre-exposure to *Sitobion avenae* altered expression of several defense responsive genes, which benefited the fungus (Meng et al., 2013). However, the role of JA and SA signaling pathways in the observed interactions is not yet known.

Considering that in agroecosystems plants are exposed to multiple attackers, the fine-tuning of their defenses is a key factor determining their fitness (Vos et al., 2013). Understanding the fundamental mechanisms and evolution of plant defenses is a crucial step for the development of sustainable control methods in agriculture. This is of great importance considering that chemical control methods are either failing, due to the ability of pests to gain resistance against them (Puinean et al., 2010; Bass et al., 2011) or are restricted due to their harmful effects on non-target beneficial organisms in the ecosystem (Simon-Delso et al., 2015).

The plant family Brassicaceae consists of many important agricultural crops including, oilseed rape (OSR), *Brassica napus*, a polyploid species result of crossing *Brassica rapa* and *Brassica oleracea* (Chalhoub et al., 2014). OSR is one of the most cultivated and profitable crops worldwide (FAOSTAT, 2018). Additionally, as with the other members of this plant family, OSR has specialized chemistry due to the production of glucosinolates (GSL) and their breakdown products that are involved in plant defenses against herbivorous insects and pathogens (Schoonhoven et al., 2005; van Dam et al., 2009). OSR is the host for the soil-borne necrotrophic pathogen *Rhizoctonia solani* (Kühn). This pathogen is characterized by great genetic variability: it is divided into 13 anastomosis groups (AG), each specialized to a certain plant host (Parameter, 1970; Ogoshi, 1987). Isolates belonging to AG 2-1 are the most pathogenic for OSR; under favorable environmental conditions they infect seedlings and cause damping off disease (Kataria and Verma, 1992; Khangura et al., 1999). Disease in this early stage leads to reduced crop establishment and consequently yield loss. Although, many studies have attempted to identify resistance traits in *B. napus*, resistant germplasm has not been identified and it remains a mystery how AG 2-1 suppresses or avoids plant defenses (Babiker et al., 2013; Sturrock et al., 2015). Oilseed rape is one of the secondary hosts of the peach-potato aphid *Myzus persicae* (Sulzer). This aphid is a particularly important pest, not only because of the direct damage it causes but also because it is the vector for more than 100 plant viruses (Blackman and Eastop, 2000). It is a very effective plant herbivore, able to gain resistance against plant defenses and even the most effective insecticides, including neonicotinoids (Bass et al., 2011). Currently it is unknown how and if *M. persicae* and *R. solani* AG 2-1 indirectly interact with each other when they share the same host-OSR and how host-plant responds to this dual attack.

The aim of the present study was, therefore, to identify if there is an interaction between herbivory by *M. persicae* and infection by AG 2-1 in *B. napus* and consequently gain a better insight into OSR defenses against two major attacking organisms. We first explored if infection with AG 2-1 had a negative effect on aphid performance, measured in relation to growth and population increase. Secondly, we examined if infestation of *M. persicae* affects the plant’s ability to defend itself against AG 2-1 infection, by assessing the disease level and quantifying fungal DNA in plants and compost. Plant performance was estimated by measuring the fresh weight. Additionally, in order to obtain a better insight of the interaction, we examined the expression of genes involved JA and SA signaling.

## Materials and Methods

### Plant Growth

*Brassica napus* plants, cultivars (cv) “Temple” and “Canard,” were grown in a controlled environment room (18°C ± 2, 12 h light: 12 h dark) for 3–4 weeks prior to use in the experiments. Seeds were sown in a mixture of 50% perlite standard (Sinclair Pro, United Kingdom) and 50% Traysubstrat (Klasmann-Deilmann GmbH, Germany) and a week later transplanted to pots (9 cm) containing Levington M3 compost (Everris Ltd., United Kingdom).

### Aphids and *Rhizoctonia solani* Inoculum

Peach-potato aphid, *M. persicae* (ISIL clone), originally obtained from a colony maintained at Rothamsted Research was reared on oilseed rape plants, cultivar “Westar” under controlled conditions (18°C ± 2, 12 h light: 12 h dark). *Rhizoctonia solani* AG 2-1 (#1934 from the isolate collection at the University of Nottingham), with known pathogenicity to OSR (Sturrock et al., 2015), was used to produce inoculum. The inoculum was grown on Potato Glucose Agar (PGA; Sigma-Aldrich, United Kingdom) for a period of 10–14 days prior to inoculation, at room temperature (18–20°C).

### Effect of AG 2-1 Infection of Plants on *Myzus persicae*

In order to assess if AG 2-1 infection affects aphid performance, one inoculum plug (5 mm) was used to inoculate each plant (10 plants from each cultivar were inoculated). The plug was cut into two equal parts and each of them was placed 1.5 cm away from the stem, opposite to each other at a depth of ∼6 cm. For the control treatment (Supplementary Table 1), plants were not inoculated. Inoculated (PA) (10 plants from each cultivar) and control (A) (10 plants from each cultivar) plants were kept in a controlled environment room with 18°C ± 2, 12 h light: 12 h dark. A week later, three alate (winged) adult aphids were placed with a fine brush on a developed leaf of each of the inoculated and control (total of 40 plants; 10 for each cultivar and 10 for each treatment) (Supplementary Table 1) plants and then a clip cage was adjusted on each leaf to ensure that the aphids were kept on the leaves. Plants were watered every 2 days. This experiment was repeated as two independent experimental replicates at different times over 6 months.

#### Aphid Performance and Reproduction

One day after infestation, adult aphids were removed and any nymphs laid were counted. If no nymphs had been laid or the adults had died, adults were replaced. The young nymphs were collected and weighed on a micro balance (Precisa XB 120A, Presica Instruments Ltd., Switzerland) and then placed back on the plants. Seven days later they were collected and weighed again in order to estimate their Mean Relative Growth Rate (MRGR) (Radford, 1967; Leather and Dixon, 1984):

$$MRGR=(InW_{2}-InW_{1})/7$$

Where W_1_ is the weight at birth and W_2_ is the weight at 7th day.

In order to estimate the intrinsic rate of population increase (r_m_), the biggest nymph (or adult) from each clip cage was placed back on the plant to lay new nymphs. For a period of a week, the number of new nymphs was recorded daily. The nymphs were removed from the plant to prevent crowding in the clip cage and to allow the adult to lay more nymphs. Intrinsic rate of population increase was estimated by the following formula, where D = the time taken from the birth of the aphid to the production of the first nymph, FD = the number of nymphs produced over a period equal to time D, 0.74 constant of Wyatt and White (1977):

$$r_{m}=0.74\left(In(FD)/D\right)$$

On the last day (14th day), the above ground plant part was collected and fresh weight was measured (Precisa 12.400 DG-FRSCS, Precisa Instruments Ltd., Switzerland) to estimate if there was any difference between treatments and varieties. All AG 2-1 inoculated plants were checked for disease symptoms.

### Effect of *Myzus persicae* on Plant Susceptibility to AG 2-1

For this experiment 3–4 week old OSR plants (cv “Canard” and “Temple”) were first infested with aphids as described previously and 3 days later, infected with AG 2-1 (AP) (Supplementary Table 1) in the same way as described above and kept in a room with controlled conditions (18°C ± 2, 12 h light: 12 h dark). For the control (P) treatment (Supplementary Table 1), plants without previous aphid infestation were inoculated with AG 2-1. Thirteen days post-inoculation (dpi) with AG 2-1, plants from both treatments were removed from the compost and the above ground plant parts were washed and disease on plant stems was visually assessed using a scale of 0–3 (0 = no symptoms, 1 = light disease; lesions occupying < 50%, 2 = moderate disease; lesions occupying 50–70%, and 3 = severe disease; lesions occupying > 70%) and weighed (Precisa 12.400 DG-FRSCS, Precisa Instruments Ltd Switzerland). For the extraction of fungal DNA, stems of each plant were cut and freeze dried (at -40°C for 4 days). Additionally, the compost was left to dry at room temperature (18°C ± 2) for a period of 6–8 days and then kept in sealed bags in a cold room (5°C ± 2) until extraction. This experiment was repeated as three independent experimental replicates at different times over 6 months.

#### Extraction of Fungal DNA From Compost

Fungal DNA from compost was extracted using a modified version of Sturrock et al. (2015). Compost from two biological replicate plants were combined into one sample (2 g) for each treatment of each variety. For homogenization, each sample was placed in a 50 ml falcon tube with three 1/4 inch ceramic balls (MP Biomedicals, United States), 15 ml of CTAB buffer (cetyltrimethylammonium bromide) and 0.45 ml of Antifoam B and homogenized using a FastPrep-24^TM^ homogeniser (MP Biomedicals, United States). DNA extraction was performed using the Wizard Magnetic DNA Purification System for Food as per manufacturer’s instructions (Promega Wizard Food Kit, Southampton, United Kingdom).

#### Extraction of Fungal DNA From Plant Material

Freeze dried stems were cut into small pieces with scissors and weighed. They were milled by adding ∼0.2 ml Lysing matrix D Bulk (MP Biomedicals, United States) to each sample tube and mechanically shaking them in a FastPrep-24^TM^ homogeniser (MP Biomedicals, United States). DNA was extracted using the method described by Ray et al. (2004) adjusting the amount of CTAB (15 ml for 2 g of plant sample) to the weight of the sample.

#### Quantification of Fungal DNA

DNA concentration of compost samples was quantified using a NanoDrop (NanoDrop^®^) at the ratios of wavelengths 260 nm/230 nm and 260 nm/280 nm, estimated as ng/μl (NanoDrop 1000 V3.8.1 software) and diluted (10^-2^ ng/μl) in TE Buffer. Concentration of DNA samples from OSR stems was quantified using a spectrophotometer (Cary 50 Probe, Varian, Australia) and diluted in TE Buffer to a standard concentration of 20 ng/μl. Prior to Real Time PCR, all DNA samples were amplified in an ITS (Internal Transcribed Spacer) PCR (White et al., 1990) to ensure that fungal DNA was present and amplifiable. For the ITS PCR a 2x MangoMix (Bioline, United Kingdom) mastermix was used and amplification was performed in a Gene Amp PCR System 9700 (Applied Biosystems, United States) programmed for: 94°C for 1 min and 15 s, followed by 35 cycles of 94°C for 15 s, 50°C for 15 s and 72°C for 45 s and finished on 72°C for 4 min and 25 s and hold at 10°C. Gel electrophoresis was carried out using 1% agarose gels stained with ethidium bromide (0.05%). PCR products were viewed on a Gel Doc 2000 system (Bio-Rad, Buckinghamshire, United Kingdom) under UV light. Real Time PCR was performed using SsoAdvanced^TM^ Universal SYBR Green Supermix (BioRad, United States) with primers specific for *R. solani* AG 2-1 (Budge et al., 2009; Supplementary Table 2). The amplification protocol was 10 min at 95°C followed by 40 cycles of 15 s at 95°C and 30 s at 64°C and then followed by 5 s at 60°C and 95°C (1000 Thermo Cycle, BioRad Laboratories Ltd., United Kingdom). Fungal DNA in samples was quantified by including six DNA standards on each PCR run. The fungal DNA standards consisted of DNA of AG2-1 (isolate #1934) used to produce six standard dilutions from 10 to 10^-5^ ng/μl. The amount of DNA was then determined by linear regression.

### Gene Expression

#### Target Genes

Target genes were selected based on their role as marker genes in the two major signaling pathways JA and SA and/or on their role in plant defenses against *M. persicae* and/or necrotrophic fungi. Five genes were selected: *LIPOXYGENASE 3* (*LOX3*), *MYC2*, *ETHYLENE RESPONSE FACTOR 1* (*ERF1)*, *NON EXPRESSOR OF PATHOGENESIS-RELATED GENES 1* (*NPR1*), *PATHOGENESIS RELATED 1* (*PR1*), and *WRKY38*. The *LOX* family acts upstream in the JA signaling pathway. MYC2 transcription factor regulates cross talk in the JA signaling pathway and defenses against herbivory (Kazan and Manners, 2013). ERF1 transcription factor is activated by coordination of ET and JA signaling pathways and regulates the expression of genes against necrotrophic pathogens (Kazan and Manners, 2013). *NPR1* is a receptor of SA and regulates the expression of *PR1* marker gene (Wu et al., 2012). WRKY transcription factors play diverse roles in basal plant defenses and *WRKY38* negatively regulates SA responses (Kim et al., 2008).

#### Collection of Samples

Based on results of experiments on the effect of *M. persicae* on plant susceptibility to AG 2-1, cv “Canard” was used for this experiment. Plants were grown, infested with *M. persicae* and inoculated with AG 2-1 as described above. Samples were collected from five different treatments: Aphid (A), pathogen (P), and aphid with pathogen (AP) and two control treatments (control 1 and control 2). We chose two time points to examine an early and a later stage of infection (earlier observations showed that at 72 h AG 2-1 hyphae reach the plant). Time points were taken at 24 h intervals to exclude the effect of circadian cycle in the expression of genes. Hence, control 1 samples were collected from plants prior to aphid infestation. Aphid samples were collected at 52 and 76 h post-infestation. Control 2 samples were collected from plants, 3 days after control 1, prior to AG 2-1 infection. Pathogen samples were collected at 72 and 120 h post-infection with AG 2-1. For the AP treatment, plants were harvested at 72 and 120 h post-AG 2-1 infection (Supplementary Figure 1).

#### RT-qPCR (Real Time Quantitative PCR)

For each sample, one fully developed leaf was collected, immediately frozen in liquid nitrogen and stored at –80°C. Five biological samples were collected for each time point/treatment. Leaf samples were ground to fine powder using a mortar and pestle and RNA was isolated using the RNeasy^®^ Plant Mini Kit (QIAGEN, Germany) and treated with DNAase I (RNase-free) (New England Biolabs, United Kingdom) following manufacturers’ instructions. For assessing the purity of the RNA, samples were analyzed by RT-PCR (program: 3 min at 95°C followed by 35 cycles of 30 s at 95°C, 30 s at 60°C, followed by 30 s at 72°C and then for 100 min at 72°C) (T100^TM^ Thermal Cycle, BioRad Laboratories Ltd., United Kingdom) and the amplifications were used to run a 1.5% agarose gel and visualized in InGenius3 with GeneSys image acquisition software (Syngene, Synoptics Ltd.). The amount of the RNA in the samples was quantified using a NanoDrop (NanoDrop^®^). First strand of cDNA synthesis was performed using qScript^TM^ cDNA SuperMIX (Quanta BioSciences, United States) as per manufacturer’s instructions and the obtained cDNA was quantified using a NanoDrop (NanoDrop^®^). RT-qPCR was carried out with three technical replicates per sample, using LuminoCt^®^ SYBR© Green qPCR Ready Mix^TM^ (Sigma-Aldrich, United Kingdom), in the following program; 1 min at 95°C followed by 60 cycles of 5 s at 95°C, 8 s at 62°C and then followed by 30 s on 72°C or in 1 min at 95°C followed by 70 cycles of 5 s at 95°C, 8 s at 50°C and then followed by 30 s on 72°C for *ERF1* gene (1000 Thermo Cycle, BioRad Laboratories Ltd., United Kingdom). For each of the target genes primers were designed and tested (Supplementary Table 2). *ACTIN* was used as reference gene (Körber et al., 2015).

### Statistical Analysis

In all experiments, 10 plants of each variety were used as biological replicates in each of the treatments. When testing the effect of AG 2-1 infection on *M. persicae*, two experimental replicates were used. When testing the effect of *M. persicae* on AG 2-1, three experimental replicates were used for disease assessments, plant fresh weight assessment and for extraction of fungal DNA from plants and two experimental replicates for the extraction of fungal DNA in compost. General ANOVA (GenStat 17th Edition) was used to detect significant interactions between treatments and varieties for MRGR of aphids, fungal DNA in compost and fresh weight. Two sample *t*-test was used for the detection of any significant differences within varieties and within treatments for disease, fungal DNA in plant stems and the intrinsic rate of population increase and fresh weight. Fungal DNA data were logarithmically transformed prior to the analysis. For the gene expression analysis, for each treatment point four to two biological replicates were used. The expression of the target genes and *ACTIN* was estimated individually for each using the technical replicates and then an average for each biological replicate in each treatment was calculated. Then the given value of each gene was expressed in relation to the value of the *ACTIN* for the same treatment. General ANOVA was used to estimate if there were significant differences between treatments and time points. In order to detect if there was an interaction between pathogen and aphid-pathogen treatment at the two tested time points, a general ANOVA with two factors (treatment and time) was performed.

## Results

### Effect of AG 2-1 Infection of Plants on *M. persicae*

**FIGURE 1:**
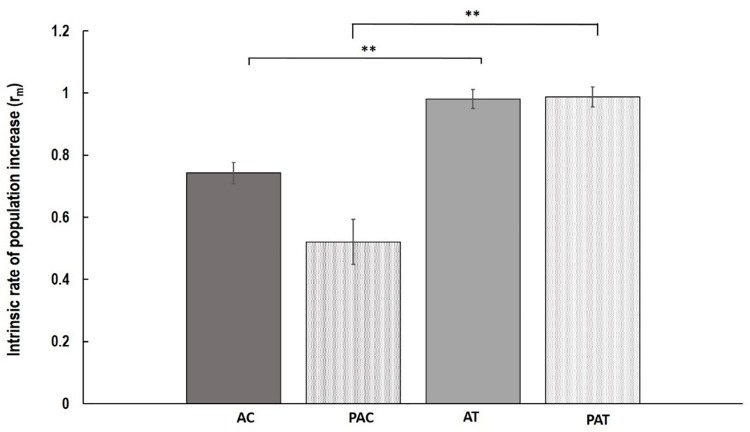
Mean of intrinsic rate of population increase (r_m_) ( ± SE) of *M. persicae* aphids on the following treatments; AC, non-inoculated control (aphid only treatment) Canard plants; PAC, Canard plants with aphids previously inoculated with AG 2-1; AT, non-inoculated control (aphid only treatment) Temple plants; PAT, Temple plants with aphids previously inoculated with AG 2-1. ^∗∗^*P* ≤ 0.01 (two-sample *t*-test).

Although, the growth of nymphs during the first week after their birth, measured as MRGR, was not different between varieties and treatments (*P* = 0.848; Table 1), significant differences were observed in population increase between varieties in both treatments (Figure 1). *Myzus persicae* adults laid more nymphs on “Temple” compared to “Canard” both for plants that had been previously inoculated with AG 2-1 (PA), 89% more nymphs (two sample *t*-test: *t* = -2.94, d.f. = 24.81, *P* = 0.0007) and for the non-inoculated control plants (A), 32% more nymphs (two sample *t*-test: *t* = -2.56, d.f. = 35, *P* = 0.0015; Figure 1). The intrinsic population growth of *M. persicae* aphids was not different between treatments either on “Canard” (two sample *t*-test: *t* = 1.39, d.f. = 25.59, *P* = 0.178) or “Temple” (two sample *t*-test: *t* = -0.08, d.f. = 34, *P* = 0.938; Figure 1). However, aphid population was increased on cv. “Temple” compared to “Canard” in both treatments (A: two sample *t*-test: *t* = -2.56, d.f. = 35, *P* = 0.015, PA: two sample *t*-test: *t* = -2.49, d.f. = 24.81, *P* = 0.007).

**Table 1:** Mean relative growth rate (MRGR) of *M. persicae* and FW of Canard and Temple under pathogen and aphid inoculation (PA) and only aphid infestation (A).

		MRGR	FW (g)
Canard	PA	0.26	7.96
	A	0.25	11.54
Temple	PA	0.20	8.47
	A	0.18	9.01
*P*_(t^∗^v)_		0.848	0.111
LSD_(t^∗^v)_		0.114	2.891


**For the comparison between treatments and varieties P value and LSD were used (ANOVA).**

No interaction was detected between treatments and varieties for the fresh weight of above ground plant parts (*P* = 0.111, ANOVA; Table 1). However, when we used two-sample *t*-test to detect if there were differences within each treatment, an effect was observed in “Canard” with AG 2-1 inoculated plants being significantly lighter (31%) compared to aphid-only control plants (two-sample *t*-test: *t* = 2.36, d.f. = 26.06, *P* = 0.026). Additionally, a significant difference was observed between the two varieties in the control plants, with “Canard” being heavier compared to “Temple” (two-sample *t*-test: *t* = 2.86, d.f. = 37, *P* = 0.003).

### Effect of *Myzus persicae* on Plant Susceptibility to AG 2-1

**FIGURE 2:**
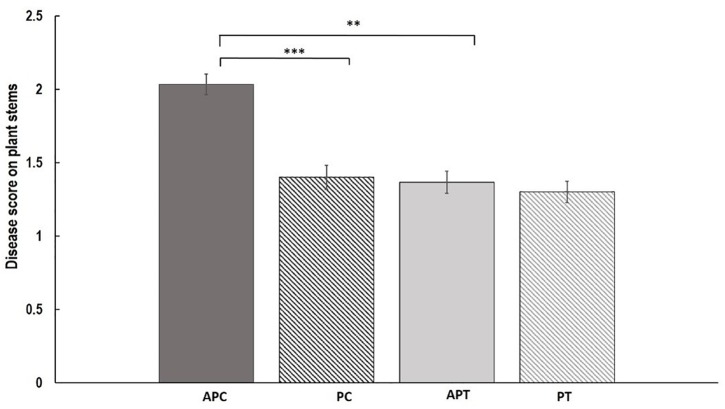
Mean of disease (±SE) caused by AG 2-1 13 dpi on OSR stems (*n* = 30) under the following treatments APC, aphid and pathogen infection on cultivar Canard; PC, only pathogen infection on cultivar Canard; APT, aphid and pathogen infection on cultivar Temple; PT, only pathogen infection on cultivar Temple. ^∗∗^*P* ≤ 0.01, ^∗∗∗^*P* ≤ 0.001 (two-sample *t*-test).

Disease assessment of stems revealed significant differences between treatments and varieties (Figure 2). Aphid infestation prior to AG 2-1 infection (AP) resulted in significantly higher disease severity (48.7% increase) in “Canard” plant stems compared to AG 2-1 only infected (P) controls (two-sample *t*-test: *t* = 3.11, d.f. = 58, *P* = 0.001; Figure 2). In addition to this, in the aphid-pathogen treatment, “Canard” plants had significantly more disease (45.2% increase) compared to “Temple” plants (two-sample *t*-test: *t* = 3.02, d.f. = 58, *P* = 0.002). Nonetheless, disease severity between the two varieties was not different in the controls (P) (two-sample *t*-test: *t* = 0.31, d.f. = 58, *P* = 0.380; Figure 2). Also, no differences were detected in disease between the two treatments in Temple plants (two-sample *t*-test: *t* = 0.47, d.f. = 58, *P* = 0.320).

**FIGURE 3:**
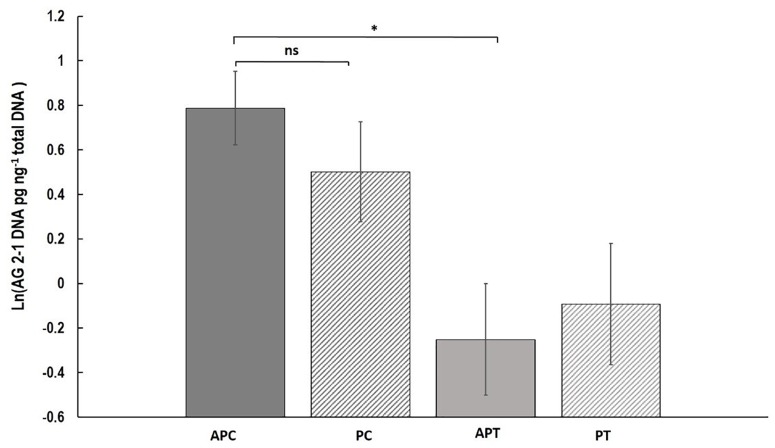
Mean of *R. solani* DNA (ln (DNA pg ng^-1^ total DNA)) (±SE) extracted from stems of OSR plants 13 dpi. Treatments; APC, aphid and pathogen infection on cultivar Canard; PC, only pathogen infection on cultivar Canard; APT, aphid and pathogen infection on cultivar Temple; PT, only pathogen infection on cultivar Temple. ^∗^*P* ≤ 0.05, (two-sample *t*-test).

Fungal DNA was significantly higher with a 56.7% increase in “Canard” plants compared to “Temple” under aphid infestation (AP) (two-sample *t*-test: *t* = 1.73, d.f. = 50.17, *P* = 0.045) but no significant differences were detected when we compared the varieties in the control (P) treatment (two-sample *t*-test: *t* = 0.85, d.f. = 57, *P* = 0.20; Figure 3). Also, no significant differences were observed between the two treatments within either “Canard” (two-sample *t*-test: *t* = 0.651, d.f. = 58, *P* = 0.306) or “Temple” (two-sample *t*-test: *t* = -0.21, d.f. = 57, *P* = 0.58; Figure 3).

The amount of AG 2-1 extracted from the compost of tested plants was not different between varieties or between treatments and there were no significant interactions between them (*P* = 0.669, LSD = 0.446; Supplementary Table 3). Similarly, the fresh weight of above ground plants was not significantly between treatments, varieties and neither was their interaction (*P* = 0.693, LSD = 1.53; Supplementary Table 3).

### Gene Expression

#### Effect of *Myzus persicae*

**FIGURE 4:**
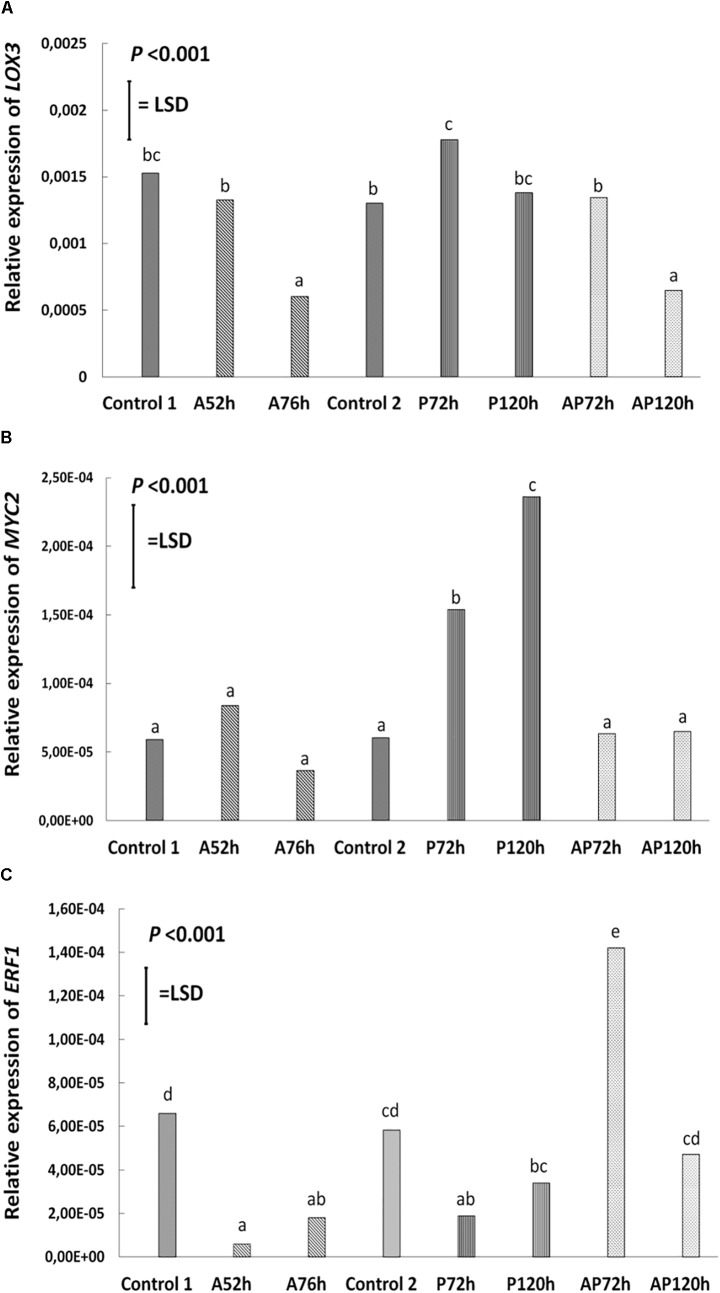
Relative expression of **(A)** *LOX3*, **(B)** *MYC2*, **(C)** *ERF1* at different treatments and time points cultivar Canard: control 1 and control 2, aphid (A); 52 and 76 h post-infestation with aphids, pathogen (P); 72 and 120 h post-inoculation with AG 2-1 and aphid and pathogen (AP); at 72 and 120 h post-infection with AG 2-1. For the comparison between the different treatments *P*-value and LSD were used, different letters indicate significant differences (ANOVA).

**FIGURE 5:**
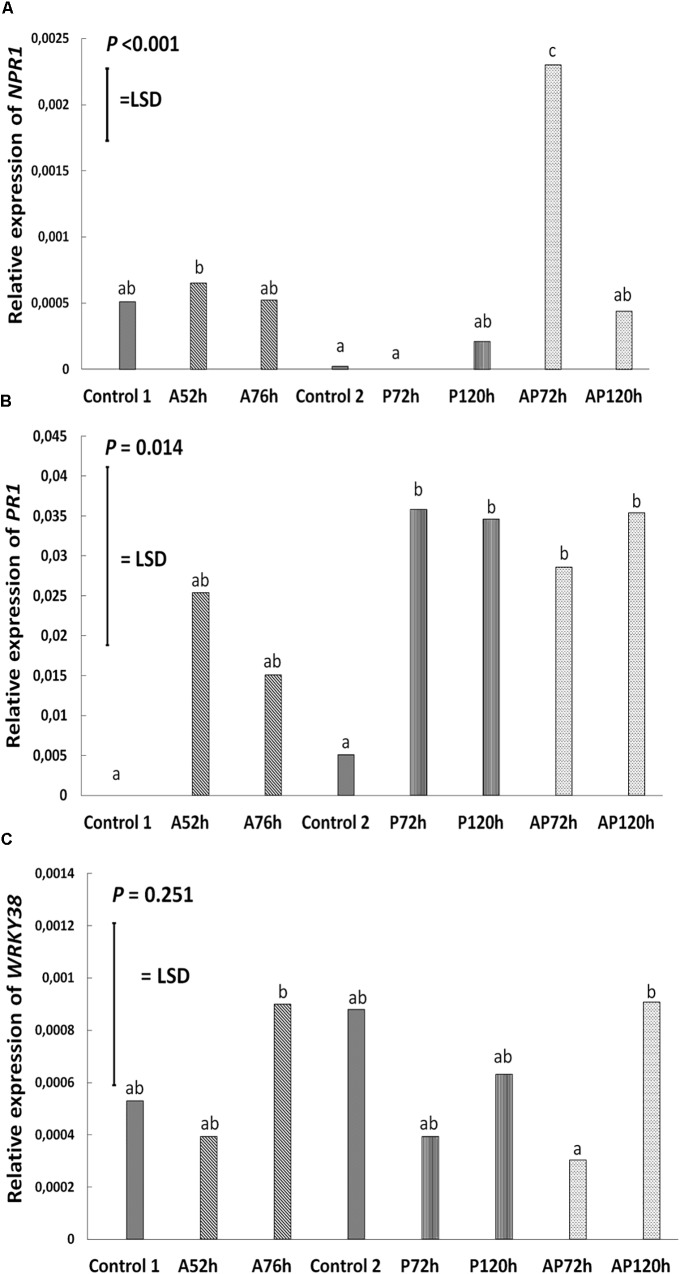
Relative expression of **(A)** *NPR1*, **(B)** *PR1*, and **(C)** *WRKY38* at different treatments and time points on cultivar Canard: control 1 and control 2, aphid (A); 52 and 76 h post-infestation with aphids, pathogen (P); 72 and 120 h post-inoculation with AG 2-1 and aphid and pathogen (AP); at 72 and 120 h post-infection with AG 2-1. For the comparison between the different treatments *P*-value and LSD were used, different letters indicate significant differences (ANOVA).

Aphid infestation induced changes in the expression of *LOX3*; the expression was downregulated after aphid infestation but was significant only 76 h after infestation compare to control 1 and control 2 (*P* < 0.001, LSD = 0.000439; Figure 4). *Myzus persicae* significantly downregulated the expression of *ERF1* both at 52 and 76 h after infestation (*P* < 0.001, LSD = 0.00002577; Figure 4) compare to the expression of the controls. The expression of *MYC2* as well as the expression of the SA marker genes, *NPR1*, *PR1*, *WRKY38*, was not significantly affected by *M. persicae* infestation (Figure 5 and Table 2).

**Table 2:** Summary of results on gene expression; relative expression of genes in relation to *ACTIN* and interaction between different treatments.

Treatment	LOX3	MYC2	ERF1	NPR1	PR1	WRKY38
C1	0.00153	0.000059	0.000066	0.00051	0	0.0005
A52	0.00133	0.000084	0.000006	0.00065	0.0254	0.0004
A76	0.00060	0.000037	0.000018	0.00052	0.0151	0.0009
C2	0.00135	0.000063	0.000058	0.00002	0.0051	0.0009
P72	0.00065	0.000065	0.000019	0	0.0358	0.0004
P120	0.00130	0.000060	0.000034	0.00021	0.0346	0.0006
AP72	0.00180	0.000154	0.000142	0.00023	0.0029	0.0003
AP120	0.00140	0.000237	0.000047	0.00044	0.0354	0.0009
*P*	<0.001	<0.001	<0.001	<0.001	0.014	0.251
LSD	0.000439	0.00006015	0.00002577	0.0005	0.0223	0.0006
**INTERACTIONS**						
C1 vs. A52	Ns	ns	Significant	ns	ns	ns
C1 vs. A76	Significant	ns	Significant	ns	ns	ns
C1 vs. C2	Ns	ns	ns	ns	ns	ns
C1 vs. P72	Ns	Significant	Significant	ns	Significant	ns
C1 vs. P120	Ns	Significant	Significant	ns	Significant	ns
C1 vs. AP72	Ns	ns	Significant	Significant	Significant	ns
C1 vs. AP120	Significant	ns	ns	ns	Significant	ns
A52 vs. A76	Significant	ns	ns	ns	ns	ns
A52 vs. C2	Ns	ns	Significant	Significant	ns	ns
A52 vs. P72	Significant	Significant	ns	Significant	ns	ns
A52 vs. P120	Ns	Significant	SIGNIFICANT	ns	ns	ns
A52 vs. AP72	Ns	ns	Significant	Significant	ns	ns
A52 vs. AP120	Significant	ns	Significant	ns	ns	ns
A76 vs. C2	Significant	ns	Significant	ns	ns	ns
A76 vs. P72	Significant	Significant	ns	ns	ns	ns
A76 vs. P120	Significant	Significant	ns	ns	ns	ns
A76 vs. AP72	Significant	ns	Significant	Significant	ns	Significant
A76 vs. AP120	Ns	ns	Significant	ns	ns	ns
C2 vs. P72	Significant	Significant	Significant	ns	Significant	ns
C2 vs. P120	Ns	Significant	Ns	ns	Significant	ns
C2 vs. AP72	Ns	ns	Significant	Significant	Significant	ns
C2 vs. AP120	Significant	ns	Ns	ns	Significant	ns
P72 vs. P120	Ns	Significant	Ns	ns	ns	ns
P72 vs. AP72	Significant	Significant	Significant	Significant	ns	ns
P72 vs. AP120	Significant	Significant	Significant	ns	ns	ns
AP72 vs. AP120	Significant	ns	Significant	Significant	ns	Significant


*To identify significance P-value and LSD (ANOVA) were used. ns indicates no significant interaction. Treatments are: C1; control 1, C2; control 2, A; aphid infestation at 52 (A52) and 76 h (A76) post-infestation, P; pathogen infection at 72 (P72) and 120 (P120) h post-infection, AP; aphid and pathogen infection at 72 (AP72) and 120 (AP 120) h post-infection.*

#### Effect of AG 2-1 Infection

Pathogen infection significantly upregulated the expression of *LOX3* at 72 h (*P* < 0.001, LSD = 0.000439; Figure 4), which returned to the basal level (similar to control 2) at 120 h post-infection (Figure 4 and Table 2). *MYC2* expression was uperegulated after the infection with AG 2-1 at 72 h and expression was further increased 120 h post-infection (*P* < 0.001, LSD = 0.00006015) (Figure 4). AG 2-1 infection downregulated the expression of *ERF1* at 72 h *P* < 0.001, LSD = 0.00002577; Figure 4), however, at 120 h the downregulation was not significantly different from control 2 (Table 2). Additionally, AG 2-1 infection led to the upregulation of PR1 at both 72 h and 120 h *P* = 0.014, LSD = 0.0223; Figure 5). However, the expression of *NPR1* and *WRKY38* was not significantly affected by AG 2-1 infection compare to control 2 (Figure 5 and Table 2).

#### Effect Simultaneous Aphid Infestation and Pathogen Infection

The presence of both aphids and pathogen (aphid-pathogen treatment) induced several changes on the expression of the tested genes; the expression of *LOX3* was significantly downregulated at 120 h (*P* < 0.001, LSD = 0.000439; Figure 4), compare to the controls and was similar to the gene expression during the aphid-only treatment at 76 h (Table 2). The expression of *MYC2* was not altered neither at 72 h nor at 120 h compare to the controls (Figure 4 and Table 2). Nonetheless, expression of *ERF1* was significantly upregulated at 72 h compared to expression levels for the two controls (*P* < 0.001, LSD = 0.00002577; Figure 4), followed by an expression similar to this of the controls at 120 h. In the presence of both *M. persicae* and AG 2-1, the expression of *NPR1* was significantly upregulated at 72h (*P* < 0.001, LSD = 0.0005; Figure 5), but at 120 h was similar to expression level of the controls. The expression of *PR1*was upregulated at both 72 h and 120 h compare to the controls (*P* = 0.014, LSD = 0.0223; Figure 5). Although, the expression of *WRKY38* at 72 h and at 120 h was not significantly different from the expression of the controls (*P* = 0.251, LSD = 0.0006; Figure 5), it was different within the treatment (AP) between the different time points at 72 and 120 h (Table 2).

#### Comparison Between the Effect of Pathogen and Aphid-Pathogen Treatments

Analysis of differences between the pathogen and aphid-pathogen treatments at 72 and 120 h, with treatment and time as different factors, revealed significant interactions for each factor (Supplementary Table 4). The expression of genes was significantly different between pathogen and aphid-pathogen treatment: in the presence of both *M. persicae* and AG 2-1, expression of *LOX3* was downregulated compared to pathogen alone (*P* = 0.009, LSD = 0.0004); similarly, for *MYC2* under the aphid-pathogen treatment, expression was downregulated compared to the pathogen treatment (*P* < 0.001, LSD = 0.0000506). The expression of both *ERF1* and *NPR1* were upregulated during the aphid-pathogen treatment (*ERF1*: *P* < 0.001, LSD = 0.00002309; *NPR1*: *P* < 0.001, LSD = 0.00046).

Additionally, differences in gene expression were observed between 72 and 120 h, with a decrease at 120 h in expression of *LOX3* (*P* = 0.013, LSD = 0.0004), *ERF1* (*P* = 0.003, LSD = 0.000023) and *NPR1* (*P* = 0.003, LSD = 0.00046). Significant interaction between treatment and time was observed for *ERF1* (*P* < 0.001, LSD = 0.00002309) and for *NPR1* (*P* < 0.001, LSD = 0.00065) (Supplementary Table 4).

## Discussion

Inducible plant defenses provide diverse strategies against a range of attackers that are activated in a species specific manner (van Loon et al., 2006). Three main phytohormonal pathways play a major role: JA, SA, and ET (Dicke and van Poecke, 2002; Dicke et al., 2003; Bari and Jones, 2009). A complex network of interactions between JA, ET, SA and other hormones such as ABA allows composition of effective plant defense strategies (Vos et al., 2013). Furthermore, pests and pathogens have co-evolved to evade or even take advantage of these defenses for their own benefit, for example by inducing SA to suppress JA. At the same time, evidence is building that plants are able to fine-tune their defenses with co-activation of phytohormone signaling pathways (Novakova et al., 2014; Li et al., 2006; Liu et al., 2016). In the present study, we investigated how *B. napus* responds to belowground infestation by AG 2-1 and aboveground herbivory by *M. persicae* and how each attacker affects the other when sharing the same host. We also tested the role of JA and SA in these interactions by analysing alterations in expression of genes involved in these signaling pathways.

Our results suggest that infection with AG 2-1 did not affect aphid performance as no significant differences were observed for both aphid growth (MRGR) and their population increase (r_m_). The peach-potato aphid is a generalist herbivore able to suppress defense mechanisms of its host plants and interfere with both SA and JA responses (De Vos et al., 2005; Thompson and Goggin, 2006). Hence, it is possible that aphids were able to overcome the defense responses induced by AG 2-1. The fact that *M. persicae* adults laid more nymphs on “Temple” plants, regardless of the treatment, compared to “Canard” implies that “Temple” served as a better host for this aphid. Perhaps these cultivars differ in their GSL profiles and therefore there is a difference in their suitability for the generalist *M. persicae*. The lack of information on the GSL profile of the tested varieties does not allow us to draw an accurate conclusion. In an interesting work from Erb et al. (2011) it was shown that maize genotypes which were less good hosts for *Spodoptera litoralis* were better hosts for *Setosphaeria turcica*. This correlates with our findings where under the presence of both AG 2-1 and *M. persicae* “Canard” plants weighed less than their controls, which implies that this variety is more susceptible to AG 2-1 infection at this growth stage. Previous screening of these varieties has shown contrasting responses regarding survival and disease during the early seedling stage (Drizou et al., 2017). In the present study, the tested plants were 3–4 weeks old during inoculation which probably alters their ability to defend themselves against AG 2-1. It is known that, AG 2-1 virulence differs based on growing stage of the plant, and is less pathogenic to older plants (Teo et al., 1988; Yitbarek et al., 1988).

When we looked at the converse situation, with *M. persicae* infestation prior to AG 2-1, we were able to detect significant differences. Although the two varieties had similar disease levels when they were exposed only to AG 2-1 (control treatment), when aphids were included in the treatment (AP) “Canard” plants had significantly more disease compared to their controls. This result implies that aphid infestation alters the ability of plants of this variety to defend themselves effectively against AG 2-1. However, this was not observed with “Temple” plants which had similar disease levels in both aphid–pathogen (AP) and P treatments. In the aphid-pathogen treatment, “Canard” plants consistently had significantly more disease compared to “Temple.”

Extracted AG 2-1 DNA from the compost did not show any significant interaction. Hence, we can conclude that the possible induced changes are not expressed as alterations in the rhizosphere. It is known that aboveground herbivory can result in translocation of nutrients and cause changes to the root exudate profile which consequently affect belowground communities (Bardgett et al., 1998). In the present study, the amount of fungal DNA was the same between treatments, so it is unlikely that alteration of exudates, if any, is stimulating AG 2-1 accumulation in the rhizosphere. Nonetheless, the extracted fungal DNA from plant stems showed a trend with the AP treatment having more fungal DNA compared to the P treatment in “Canard,” although this was not a statistically significant difference. It therefore appears that the main reason for increased disease in “Canard” under the aphid-pathogen treatment was the induced changes by *M. persicae* making the plant more susceptible to the disease rather than the actual amount of AG 2-1 in the plant. Between the two varieties, “Canard” tended to have more fungal DNA and consequently more disease compared to “Temple.” Although, the difference between the two varieties was not statistically significant within the control treatment, in the AP treatment “Temple” had significantly less fungal DNA compared to “Canard.”

In order to gain a better insight into which factors altered “Canard” response to AG 2-1 under aphid infestation, we decided to examine the expression of marker genes for JA and SA signaling. *M. persicae* induces both SA- and JA- related defenses. Moran and Thompson (2001) showed that *M. persicae* infestation in Arabidopsis resulted in transcription of *PR1* and *LOX2* but not *LOX1* (Moran and Thompson, 2001). Moreover, although herbivory by *M. persicae* did not alter SA, JA and ET levels, it induced changes in expression of 2,181 genes in Arabidopsis, including consistent twofold changes in expression of *PR1* but not *PDF1.2* (a marker gene for JA and ET, downstream of the ERF transcription factor) (De Vos et al., 2005). However, in *Brassica oleracea*, *M. persicae* did not induce the expression of *BoLOX*, a cloned *LOX* gene from *B. oleracea* sharing similarities with *AtLOX2* in *Arabidopsis thaliana* and *BnLOX2fl* in *B. napus* (Zheng et al., 2007). In the present work, *M. persicae* downregulated expression of *LOX3* at 76 h after infestation, as well as expression of *ERF1* at 52 and 76 h but expression of other genes was not significantly different compared to control 1, although there were small differences in the actual amounts of the genes between the different tested times. It is tempting to speculate that *M. persicae* induced changes suppress or overcome defenses in *B. napus* such as *LOX3*. In this regard, the peach-potato aphid is known to have the ability to deploy host plant defenses for its own benefit by effectors in saliva secretions (Elzinga et al., 2014); it is suggested that depending on its host plant, *M. persicae* changes the expression of these effectors to overcome defenses (such as GSL compounds of *Brassica* species) to enable colonization of the plant. Therefore, it might be the case that similar activation of salivary effectors resulted in the observed gene expression in the present study.

There is limited work focusing on the molecular interaction between *R. solani* and its hosts. In a recent study authors discovered that VOCs from *R. solani* AG 2-2 IIB primed *A. thaliana* plants for improved growth but did not affect disease resistance, however, it improved *Mamestra brassicae* caterpillar performance above ground (Cordovez et al., 2017). To understand the underling molecular mechanism of these observations they performed wide transcriptome analysis and found that AG 2-2 IIB VOCs triggered the upregulation of genes involved with auxin and ABA but downregulated ET- and JA- responsive genes, indicating that the observed growth-promoting effect by VOCs is facilitated by other signaling pathways (Cordovez et al., 2017).

Screening of different Arabidopsis ecotypes and mutants in signaling pathways with AG 8 and AG 2-1 by Foley et al., revealed that resistance to AG 8 and susceptibility to AG 2-1 was not related to the major signaling pathways (Foley et al., 2013). The authors argued that the final outcome of the interaction between *Arabidopsis* and these AGs should be due to the combination of JA, SA, and ET (Foley et al., 2013). Additionally, in the same work both AGs induced changes in the expression of several genes including several *PR* genes (with only AG 2-1 to induce *PR1*) and transcription factors. Although NAPDH oxidases played a key role for resistance to AG 8, this was not the case with AG 2-1 which probably overcomes host defenses (Foley et al., 2013). Another study in *Arabidopsis* (Perl-Treves et al., 2004) showed that plants respond to *R. solani* infection by inducing the glutathione *S*-transferase *GSTF8* gene promoter independently from SA signaling and this induction was only mediated by the least pathogenic AG 8. AG 2-1 did not induce the promoter and actually killed the plants. The authors stated that AG 2-1 might be able either to escape or suppress plant defense mechanism (Perl-Treves et al., 2004). From those two studies, it becomes evident that AG 2-1 is a particularly interesting pathogen which possibly has an ability to manipulate plant host defenses.

In the present study AG 2-1 induced the expression of three genes: *LOX3* 72 h post-infection, *MYC2* and *PR1* at both 72 and 120 h after infection and downregulated the expression of *ERF1* at 72h after infection. MYC is known to upregulate wounding/herbivory induce genes such as *LOX* (Lorenzo et al., 2004; Dombrecht et al., 2007). Additionally, *MYC2* is known to downregulate the expression of the ERF branch of the JA signaling pathway that is responsible for defense against necrotrophic pathogens (Dombrecht et al., 2007) and Foley et al. showed that ERF transcription factors were induced by *R. solani* (Foley et al., 2013). Consequently, the present study confirms the well-established cross-talk between the MYC2 and the ERF branch of JA pathway (Vos et al., 2015). However, it is surprising that instead of the expected induction of *ERF1* and downregulation of *MYC2* we observed the opposite effect. Nonetheless, we have to take into account that this study is in *B. napus* and not in *Arabidopsis* hence different interactions in the signaling pathways may occur. Additionally, it might be the case that AG 2-1 actually induces the expression of *MYC2* and this way interferes with the ERF branch of JA and escapes an efficient plant defense against necrotrophic fungi. There is some evidence supporting this hypothesis from studies with other necrotrophic fungi: *Alternaria brassicola* is known to deploy defenses of the susceptible host *Brassica juncea* and induce SA-regulated responses and block JA responses, while in the resistant *Sinapis alba* induction of ABA leads to JA response and efficient plant resistance (Mazumder et al., 2013).

In another pathosystem, *Sclerotinia sclerotiorum* induced responses in *B. napus* that were related to both JA and SA signaling pathways; there was an increase in the level of plant hormones and the expression of marker genes including *LOX3* and *PR1* (Novakova et al., 2014). Moreover, the WRKY family of transcription factors is known to have a role in basal plant defenses and AG 2-1 and AG 8 are known to induce the expression of this family in Arabidopsis (Foley et al., 2013). Here the expression of *WRKY38* was similar and not significantly different from the controls. *WRKY38* has been shown to negatively regulate SA-related defense and result in susceptibility of Arabidopsis to *Pseudomonas syringae* bacteria (Kim et al., 2008). However, the induced expression of *PR1* in our experiments contrasts with that, so it seems that either this effect is not present in our study system or that unknown interactions within the signaling pathways resulted in this outcome.

Furthermore, when OSR plants were exposed to both attackers, we found that although *LOX3* expression was similar to controls 72 h post-inoculation, it was downregulated at 120 h, whereas *MYC2* had no significant induction at either 72 or 120 h post-inoculation. However, the expression of *ERF1* was significantly upregulated 72 h post-inoculation and returned to basal levels at 120 h. Similarly, expression of *NPR1* was significantly increased at 72 h but was reduced and was similar to the control at 120 h post-inoculation. Expression of *PR1* increased at both examined time points and *WRKY38* had an increase only at 120 h. So there was a differentiation in gene expression when plants were under dual attack compared to when attacked by aphids or pathogen alone. As our main aim was to understand how *M. persicae* affects plant responses to AG 2-1, we compared the P treatment with the AP treatment; the expression of *LOX3* and *MYC2* was significantly downregulated by the AP treatment compared to the P treatment at both examined times indicating that *M. persicae* induces changes that suppress the expression of AG 2-1 induced genes. Considering our hypothesis that AG 2-1 increases *MYC2* in order to block the ERF branch and overcome plant defenses, we would expect that in the presence of aphids, disease symptoms would be reduced and not increased. Therefore, it seems that the interactions that are taking place and shape the final outcome are more complicated. It is unclear how aphid infestation in combination with AG 2-1 infection resulted in the upregulation of *ERF1* at 72 h and it is possible that ET, as co-regulator for the expression of this gene, is also a crucial factor in the shaping of this effect. However, the increased expression of *NPR1* 72 h post-inoculation under dual attack is also interesting as this gene is known to be a SA receptor regulating the expression of many defense-induced genes (Wu et al., 2012). Taking these results together, we can conclude that during dual attack, *M. persicae* infestation suppresses JA-responsive genes and promotes expression of SA- related genes through unknown interactions which make *B. napus* more susceptible to AG 2-1.

## Conclusion

This work provides, for the first time, information about the interaction between two major enemies of OSR: *M. persicae* and *R. solani* AG 2-1. Our data show that aphid infestation induced changes in OSR that increased susceptibility of “Canard” plants to AG 2-1 infection, likely due to the suppression of JA signaling pathway. Additionally, we found that *R. solani* AG 2-1 induced the activation of both JA- and SA-responsive genes. However, due to the complexity between the signaling pathways we cannot draw any further conclusion. Future studies should focus on the transcriptomic analysis of marker genes as well as the quantification of all major plant hormones and test the possible role of ET and ABA in the interaction.

## Data Availability Statement

All data generated or analyzed during this study are included in the manuscript and the Supplementary Material.

## Author Contributions

Practical work was designed and performed by FD under the supervision of NG, RR, and TB. The manuscript was composed by FD with the contribution of NG, RR, and TB.

## Conflict of Interest Statement

The authors declare that the research was conducted in the absence of any commercial or financial relationships that could be construed as a potential conflict of interest.

## Abbreviations

A: aphid infested plants
AG: anastomosis group
AP: aphid infested plants followed by pathogen inoculation
cv: Cultivars
dpi: days post-inoculation
FW: fresh plant weight
h: hours
MRGR: Mean Relative Growth Rate
OSR: oilseed rape
PA: pathogen infected plants followed by aphid infestation
P: pathogen inoculated plants
r_m_: population increase
ITS: Internal Transcribed Spacer
