# Supplementary material for: Infestation by Myzus persicae Increases Susceptibility of Brassica napus cv. “Canard” to Rhizoctonia solani AG 2-1
Source: Front Plant Sci. 2018 Dec 21;9:1903. doi: 10.3389/fpls.2018.01903 (PMC6308127; doi:10.3389/fpls.2018.01903)
Supplement: Supplementary file 1 [file Table_1.DOCX]

| **Abbreviation** | **Treatment** |
| --- | --- |
| AC | Aphid infestation only (aphid control) on cultivar Canard |
| PAC | Pathogen infection followed by aphid infestation on cultivar Canard |
| APC | Aphid infestation followed by pathogen inoculation on cultivar Canard |
| PC | Pathogen infection only (pathogen control) on cultivar Canard |
| AT | Aphid infestation only (aphid control) on cultivar Temple |
| PAT | Pathogen infection followed by aphid infestation on cultivar Temple |
| APT | Aphid infestation followed by pathogen inoculation on cultivar Temple |
| PT | Pathogen infection only (pathogen control) on cultivar Temple |

**Supplementary Table 1**: List of abbreviations of treatments for the experiments on the effect of AG 2-1 infection of plants to *M. persicae* and the effect of *M. persicae* on plant susceptibility to AG 2-1.
