# Supplementary material for: Infestation by Myzus persicae Increases Susceptibility of Brassica napus cv. “Canard” to Rhizoctonia solani AG 2-1
Source: Front Plant Sci. 2018 Dec 21;9:1903. doi: 10.3389/fpls.2018.01903 (PMC6308127; doi:10.3389/fpls.2018.01903)
Supplement: Supplementary file 2 [file Table_2.DOCX]

**Supplementary Table 2** Sequence of forward and reverse primers for the ribosomal ITS1 region of the *R. solani* used in ITS and RT-PCR in the compost extractions, the target genes and *ACTIN* (reference gene)

| **Gene** | **Forward primer** | **Reverse primer** |
| --- | --- | --- |
| ITS1 | 5’-CTTCCTCTTTCATCCCACACA-3’ | 5’-TGAGTAGACAGAGGGTCCAATAACCTA-3’ |
| *LOX3* | 5’-GGCCTTACCCTAGACGGTGT-3’ | 5’-TTCAAATTGCTCGTCTCGTG-3’ |
| *ERF1* | 5’-GTTTGAAAGCGCCGAAGAAG-3’ | 5’- CGAAAGCGACTCTTTAACTCTCTC-3’ |
| *MYC2* | 5’-TGCGTGAGCTCAATTCTTTG-3’ | 5’-GCTCTGTGTCATCGAAACCA-3’ |
| *NPR1* | 5’-AGGGGATATACGGTGCTTCA-3’ | 5’-GAGAGCCGTTCTACCTTCCA-3’ |
| *PR1* | 5’-ATGTCAACGCTCACAACCAA-3’ | 5’-TCTTAGTCGGTCGGCGTAGT-3’ |
| *WRKY38* | 5’-GGACCAGTACCGTGGGATAA-3’ | 5’-GGGATAACCGGTGACGATAA-3’ |
| *ACTIN* | 5’- TCAGGCCGTTCTTTCTCTTTAC-3’ | 5’-GAGCATAACCCTCGTAGATTGG-3’ |
