# Supplementary material for: Infestation by Myzus persicae Increases Susceptibility of Brassica napus cv. “Canard” to Rhizoctonia solani AG 2-1
Source: Front Plant Sci. 2018 Dec 21;9:1903. doi: 10.3389/fpls.2018.01903 (PMC6308127; doi:10.3389/fpls.2018.01903)
Supplement: Supplementary file 3 [file Table_3.DOCX]

**Supplementary Table 3** Fungal DNA (log10 (DNA pg ng^-1^ of total DNA)) extracted from compost and Fresh weight (F.W.) of Canard and Temple under treatment with aphids and pathogen (AP) and pathogen only (P). For the comparison between treatments and varieties *P* value and LSD (for treatment-variety interaction) were used (ANOVA).

|  | | **DNA ( pg ng^-1^)** | **F.W (g)** |
| --- | --- | --- | --- |
| Canard | AP | 2.16 | 11.98 |
|  | P | 2.00 | 11.30 |
| Temple | AP | 2.06 | 11.45 |
|  | P | 2.16 | 11.20 |
| *P*_(t*v)_ |  | 0.669 | 0.693 |
| LSD_(t*v)_ |  | 0.446 | 1.537 |
