# Supplementary material for: Infestation by Myzus persicae Increases Susceptibility of Brassica napus cv. “Canard” to Rhizoctonia solani AG 2-1
Source: Front Plant Sci. 2018 Dec 21;9:1903. doi: 10.3389/fpls.2018.01903 (PMC6308127; doi:10.3389/fpls.2018.01903)
Supplement: Supplementary file 4 [file Table_4.docx]

**Supplementary Table 4** Relative expression of of *LOX3*, *MYC2*, *ERF1*, *NPR1*, *PR1* and *WRKY38* for treatment (P; pathogen infection, AP; aphid and pathogen), time (72 and 120 hours post infection) and the interaction treatment with time (P72 h, P120 h, AP72 h, AP120 h). For the comparison between treatments, time and their interaction, *P*_(treatment)_ value and LSD_(treatment),_ *P*_(time)_ value and LSD_(time)_ and *P*_(treatment*time)_ value and LSD_(treatment*time)_ respectively were used (General ANOVA with two factors).

|  | |  | **LOX3** | **MYC2** | **ERF1** | **NPR1** | **PR1** | **WRKY38** |
| --- | --- | --- | --- | --- | --- | --- | --- | --- |
| treatment | P | | 0.00158 | 0.00013 | 0.000026 | 0.0001 | 0.0352 | 0.00051 |
|  | AP | | 0.00099 | 0.00006 | 0.000095 | 0.00137 | 0.0320 | 0.00061 |
| time (hours) | 72 | | 0.00160 | 0.00011 | 0.000080 | 0.00115 | 0.0322 | 0.00035 |
|  | 120 | | 0.00102 | 0.00015 | 0.000041 | 0.00032 | 0.0350 | 0.00077 |
| treatment*time | P72 | | 0.00178 | 0.00015 | 0.000019 | 0 | 0.0358 | 0.00039 |
|  | P120 | | 0.00138 | 0.00024 | 0.000034 | 0.00021 | 0.0346 | 0.00063 |
|  | AP72 | | 0.00135 | 0.00006 | 0.000142 | 0.00230 | 0.0286 | 0.00030 |
|  | AP120 | | 0.00065 | 0.00006 | 0.000047 | 0.00044 | 0.0354 | 0.00091 |
|  | *P*_(treatment)_ | | 0.009 | <0.001 | <0.001 | <0.001 | 0.74 | 0.644 |
|  |  | | 0.0004 | 0.0000506 | 0.00002309 | 0.00046 | 0.02051 | 0.000441 |
|  | *P*_(time)_ | | 0.013 | 0.096 | 0.003 | 0.003 | 0.769 | 0.059 |
|  | LSD_(time)_ | | 0.0004 | 0.0000506 | 0.00002309 | 0.00046 | 0.02051 | 0.000441 |
|  | *P*_(treatment*time)_ | | 0.426 | 0.106 | <0.001 | <0.001 | 0.675 | 0.373 |
|  | LSD_(treatment*time)_ | | 0.0006 | 0.000715 | 0.00003266 | 0.00065 | 0.02901 | 0.000624 |
