# Supplementary figures and images for: Infestation by Myzus persicae Increases Susceptibility of Brassica napus cv. “Canard” to Rhizoctonia solani AG 2-1
Source: Front Plant Sci. 2018 Dec 21;9:1903. doi: 10.3389/fpls.2018.01903 (PMC6308127; doi:10.3389/fpls.2018.01903)

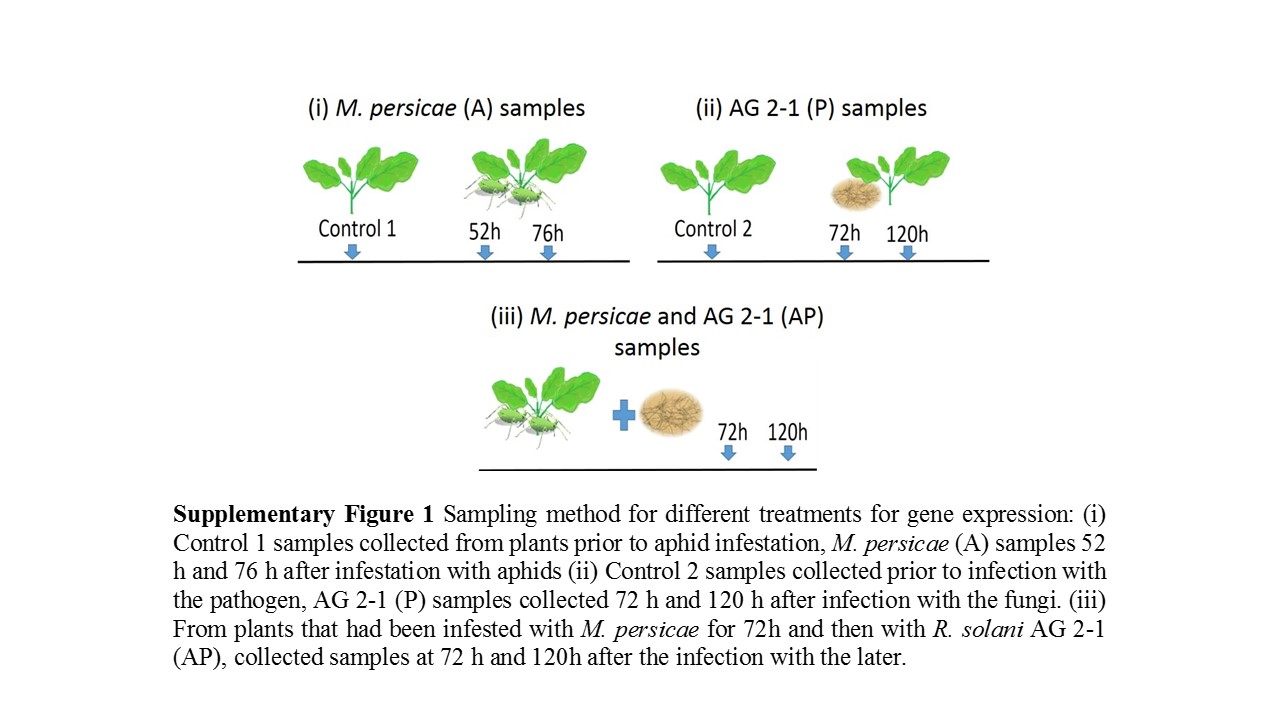

Supplement: Supplementary file 5 [file Image_1.JPEG]
